# Photovoltaic activity of electrodes based on intact photosystem I electrodeposited on bare conducting glass

**DOI:** 10.1007/s11120-020-00722-1

**Published:** 2020-02-20

**Authors:** Sebastian Szewczyk, Rafał Białek, Gotard Burdziński, Krzysztof Gibasiewicz

**Affiliations:** Faculty of Physics, Adam Mickiewicz University in Poznań, ul. Uniwersytetu Poznańskiego 2, 61-614 Poznań, Poland

**Keywords:** Photosystem I, Cyanobacterium *Synechocystis* sp. PCC 6803, Femtosecond-transient absorption, Photovoltaics, FTO conducting glass, Photoelectrochemical measurements

## Abstract

We demonstrate photovoltaic activity of electrodes composed of fluorine-doped tin oxide (FTO) conducting glass and a multilayer of trimeric photosystem I (PSI) from cyanobacterium *Synechocystis* sp. PCC 6803 yielding, at open circuit potential (OCP) of + 100 mV (vs. SHE), internal quantum efficiency of (0.37 ± 0.11)% and photocurrent density of up to (0.5 ± 0.1) µA/cm^2^. The photocurrent measured for OCP is of cathodic nature meaning that preferentially the electrons are injected from the conducting layer of the FTO glass to the photooxidized PSI primary electron donor, P700^+^, and further transferred from the photoreduced final electron acceptor of PSI, F_b_^−^, via ascorbate electrolyte to the counter electrode. This observation is consistent with preferential donor-side orientation of PSI on FTO imposed by applied electrodeposition. However, by applying high-positive bias (+ 620 mV) to the PSI-FTO electrode, exceeding redox midpoint potential of P700 (+ 450 mV), the photocurrent reverses its orientation and becomes anodic. This is explained by “switching off” the natural photoactivity of PSI particles (by the electrochemical oxidation of P700 to P700^+^) and “switching on” the anodic photocurrent from PSI antenna Chls prone to photooxidation at high potentials. The efficient control of the P700 redox state (P700 or P700^+^) by external bias applied to the PSI-FTO electrodes was evidenced by ultrafast transient absorption spectroscopy. The advantage of the presented system is its structural simplicity together with in situ-proven high intactness of the PSI particles.

**Electronic supplementary material:**

The online version of this article (10.1007/s11120-020-00722-1) contains supplementary material, which is available to authorized users.

## Introduction

Solar cells are desirable but still relatively expensive devices for the electrical energy production. Therefore, many attempts have been undertaken to discover new technologies of solar energy conversion. Among them, utilization of biological light-converting systems, in particular those involved in natural photosynthesis, is often postulated to be the promising direction of research (Kornienko et al. 2018).

Photosystem I (PSI), a photosynthetic pigment–protein complex (Brettel 1997; Brettel and Leibl 2001; Jordan et al. 2001; Amunts et al. 2007; Qin et al. 2015, 2019), is a natural and very efficient biological converter of light energy into the energy of electrical current. Its quantum efficiency of absorbed photon to electron conversion exceeds 99% and the voltage generated after light-induced charge separation (electron transfer) between the primary and final redox cofactors inside PSI is ~ 1 V. Therefore, PSI is often used as a photosensitive component of different types of working photoelectrodes being a part of prototype biosolar cells (Robinson et al. 2018; Musazade et al. 2018; Friebe and Frese 2017; Milano et al. 2019).

The structure and organization of PSI complex is somewhat different in various photosynthetic organisms (Fromme and Grotjohan 2008; Nelson and Junge 2015). It always contains PSI core, which is largely similar in all organisms, and much more diverse external antennas (LHCI)—present in algae and plants but not in cyanobacteria. Instead, in cyanobacteria, the PSI cores form regular trimers. The structure of monomeric cyanobacterial PSI core from *Thermosynechococcus elongates *(Jordan et al. 2001), which is a model for the cyanobacterium studied in this work, reveals in total 127 cofactors (96 chlorophylls *a*, 2 phylloquinones, 3 iron–sulfur clusters, 22 carotenoids, and 4 lipids) and 12 protein subunits. Six chlorophylls (Chl) plus two phylloquinons are grouped in two quasi-symmetric branches and together with 3 iron–sulfur clusters form reaction center (RC), where a series of electron transfer reactions takes place following the photon absorption by the PSI complex. Two strongly interacting Chls form P700, the dimer that is often regarded as the primary electron donor. After direct photoexcitation of P700 or its excitation following the energy transfer from any other antenna Chl excited by light, electron is transferred from P700 via all other RC redox cofactors (Chls, phylloquinons, and Fe–S clusters) and reaches the final acceptor, the iron–sulfur cluster F_b_. As a result, a long-lived charge-separated state P700^+^F_b_^−^ (or P^+^F_b_^−^) is formed within < 1 µs (Brettel and Leibl 2001). In isolated PSI, this state may be depopulated either by transferring the electron from F_b_^−^ and the hole from P700^+^ to exogenous mediator or electrode, or by a competitive dissipative internal charge recombination occurring on the hundreds of millisecond time scale (Vassiliev et al. 1997; Kurashov et al. 2018).

What differs various PSI-containing photoelectrodes described in the literature is: (1) the substrate on which PSI is deposited, (2) the method of PSI immobilization on the substrate, (3) the way by which the electric contact between PSI and the substrate as well as between PSI and the counter electrode is achieved, (4) the species from which the PSI is extracted and the type of the PSI preparation. The electrode substrates used for PSI immobilization are gold (Ciesielski et al. 2008, 2010a, b; Mukherjee et al. 2010; Stieger et al. 2016a), graphene (Gunther et al. 2013; Feifel et al. 2015), titanium dioxide (Mershin et al. 2012; Gordiichuk et al. 2014; Shah et al. 2015; Yu et al. 2015; Gizzie et al. 2015), or a conducting glass, FTO or ITO (Indium-doped Tin Oxide) (Ocakoglu et al. 2014; Stieger et al. 2016b; Szewczyk et al. 2017, 2018). The substrate may have a flat surface or a porous structure to increase the number of attached proteins and thus to maximize the photocurrent. Immobilization of the PSI on the substrate is reached most often by drop-casting of the PSI solution followed by solvent evaporation, or by encapsulation of the PSI in polymer attached to the substrate. The PSI complexes exchange the electrons with the substrate directly (in the case of monolayers) or indirectly via the redox mediators. The PSI particles are extracted from cyanobacteria, algae, and higher plants and then prepared with full or truncated antenna system. Photocurrent generated from 1 cm^2^ of the mentioned above PSI-containing electrodes ranges from below 1 µA to hundreds of µA, although the intact operation of PSI in these systems is usually assumed rather than demonstrated. In fact, it has been recently demonstrated that in the cyanobacterial PSI immobilized in a trehalose glassy matrix as much as 90% of forward electron transfer was arrested and back electron transfer was observed instead, and only 10% of the electrons excited at the PSI primary donor reached the final PSI electron acceptor (Kurashov et al. 2018). Harsh treatment of the PSI in some systems may inactivate even higher percentage of the proteins. For example, application of Co^II^/Co^III^ electrolyte dissolved in 40% acetonitrile (Mershin et al. 2012) or iodide electrolyte dissolved in 100% acetonitrile (Yu et al. 2015) may denature PSI and extract pigments from it. Thus, the high anodic photocurrent reported in these papers (of the order of 1 mA/cm^2^) may come from excitation of the isolated pigments interacting directly with the semiconducting substrates (TiO_2_ or ZnO) in a way described for dye-sensitized solar cells (DSSC; O’Regan and Grätzel 1991) in which the electrons are injected from the excited states of pigments to the conduction band of a semiconductor. Another source of the anodic photocurrent may be excited chlorophylls (Chls) physically attached to the PSI complex but energetically uncoupled from the PSI reaction center. Peripheral PSI Chls are very prone to such uncoupling (Melkozernov et al. 2004; Szewczyk et al. 2020). Such excited Chls may also inject electrons to the substrate. To sum up, both low and high efficiencies of the PSI-containing electrodes may be caused by functionally non-intact PSI complexes. Therefore, it is very important to prove that intact PSI complexes underlie the photovoltaic activity of electrodes.

Cyanobacterial PSI immobilized directly on FTO conducting glass has recently been shown by ultrafast optical spectroscopy to preserve excitation quenching properties very similar to those of the native PSI in solution (Szewczyk et al. 2017, 2018). The detailed analysis has shown that the observed modest acceleration of the excitation decay in immobilized PSI (by a few ps) may be assigned to the crowding effect similar to that reported for aggregated LHCII (Ruban et al. 2007). Additionally, interaction between peripheral chlorophylls (Chls) from neighboring PSI monomers (but not trimers) resulted in increased number of red Chls from 3 to 6 per monomeric PSI. Still, these minor effects did not question the overall natural functioning of the PSI in this semi-artificial system at the level of primary reactions: excitation trapping by the reaction center and the primary charge separation.

In the following, we present photovoltaic data obtained from the system containing simple PSI-FTO photoelectrode identical to that one described in Szewczyk et al. (2017, 2018) and propose a mechanism of the anodic and cathodic photocurrent generation in this system. The advantage of this system is the functionality of immobilized PSI proven to be highly intact at the level of excitation decay and charge separation. Moreover, for the first time, we evidence, using ultrafast time-resolved transient absorption spectroscopy, a possibility of a full control of the P700 redox state by an external bias applied to the PSI-containing electrode.

## Materials and methods

### Sample preparation

**Fig. 1:**
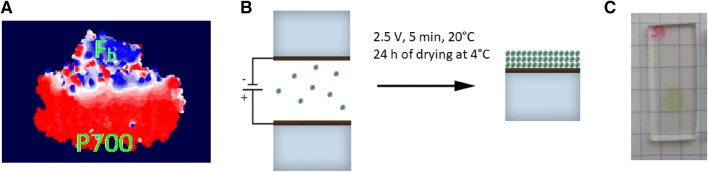
**a** Theoretical distribution of surface charge on monomeric PSI from cyanobacterium *Thermosynechococcus elongatus* estimated using Poisson–Boltzmann Surface Area method (SwissPdbViewer, pH 7.0). Red—negatively charged P700-containing lumenal or donor side of PSI; blue—positively charged F_b_-containing stromal or acceptor side of PSI. **b** Outline of the electrodeposition method of PSI (green dots) on FTO-layered (black line) glass (blue rectangle). **c** Picture of PSI-FTO electrode

*Synechocystis* sp. PCC 6803 cell cultures were grown and trimeric PSI particles were isolated and immobilized on the FTO glass as described in Szewczyk et al. (2017). Shortly, immobilization was realized by drop-casting of PSI solution containing 10 mM Bis Tris pH 7.0 (typically, 30–40 µl of absorbance or optical density OD_679nm,1 cm_ ≈ 1) previously dialyzed to decrease the concentration of detergent used for isolation (*n*-dodecyl *β*-d-maltoside, *β*-DM). The drop-casting was followed by 5-min application of positive potential (+ 2.5 V) to the FTO glass in reference to the counter electrode (also the FTO glass). Under these conditions, the PSI particles with surface charges distributed in a way shown in Fig. 1a preferentially migrated to the positively charged lower FTO electrode (Fig. 1b) and oriented with the P700-side facing the surface of FTO. To provide further evidence that such an orientation of PSI on FTO is caused by the electrical field we performed a control electrodeposition with the inversed polarity of the electrical field: when the positive voltage was applied to the upper FTO electrode, the majority of the PSI proteins migrated upward, against the gravitation and attached to the upper FTO electrode (Fig. 1b). After the electrodeposition, the solvent was evaporated at 4 °C overnight. The final estimated absorbance of the dry PSI multilayer on the FTO glass (Fig. 1) at *Q*_y_ band maximum (at ~ 679 nm) was *A*_679 nm_ = 0.030 ± 0.005 and the approximated area of such multilayer was 0.25 cm^2^. The average number of monolayers, $n_{{{\text{PSI}}}} ,$ forming the PSI multilayer was estimated from Eq. 1 to be ~ 5 assuming a dense PSI packing on the FTO (without free spaces between), the diameter of single monomeric PSI complex of *d* = 12 nm (estimated from the structure in Jordan et al. 2001) and molar extinction coefficient of Chl *a* in PSI of *ε*_679 nm_ = 57 000 M^−1^ cm^−1^ (Müh and Zouni 2005). *N*_*A*_ is the Avogadro constant, and *m*—the number of Chls *a* per monomeric PSI (96).

1 $$n_{{{\text{PSI}}}} = \frac{{A_{{679\;{\text{nm}}}} N_{{{\text{A}} }} d^{2} }}{{\varepsilon_{{679\;{\text{nm}}}} m}}$$

The PSI absorbance on FTO was measured using a Hitachi U-2800A spectrophotometer. Its value was corrected by subtraction of the absorbance of the FTO glass. The real number of monolayers was varying from spot to spot due to non-uniform distribution of PSI on the substrate observed under optical microscope over a single sample. The inhomogeneities were of submillimeter sizes and were characterized by various intensities of the green color. However, this inhomogeneity should have been averaged out in the femtosecond-transient absorption experiments by the continuous moving of the sample (see below).

We have checked that PSI monomers are equally efficiently immobilized on the FTO slide as trimers and similar photocurrents were obtained from both types of samples.

### Photoelectrochemical measurements

**Fig. 2:**
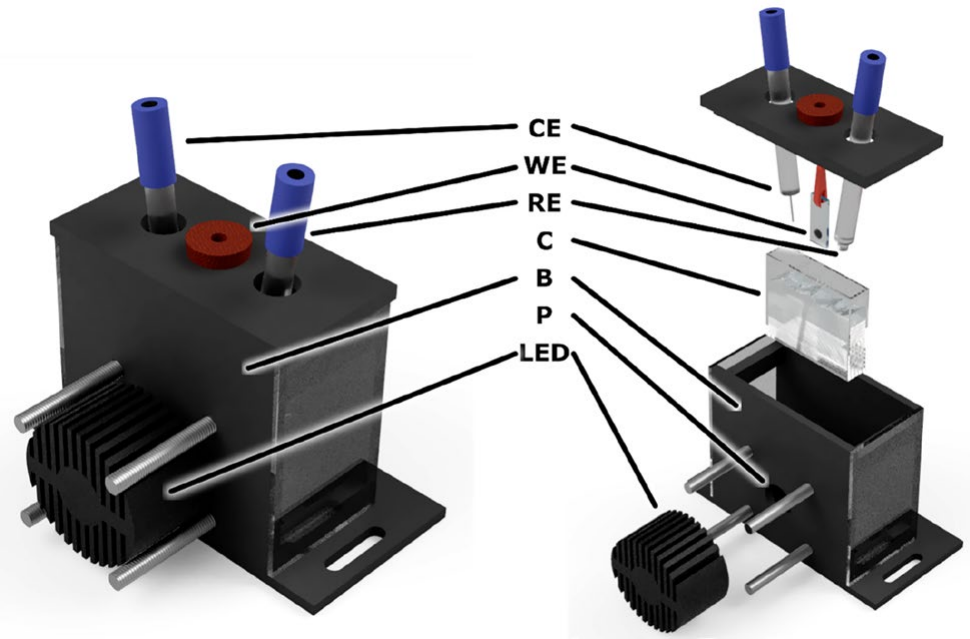
Home-build three-electrode spectroelectrochemical cell for photocurrent measurements: assembled (left) and exploded (right). WE—working electrode composed of FTO conducting glass covered with PSI multilayer; CE—Pt counter electrode; RE—Ag/AgCl reference electrode; C—cuvette filled with electrolyte; B—black-walled box; P—port for the LED illuminating the WE; LED—Light-Emitting Diode with a radiator used as an illumination source

Photocurrent measurements were performed in a specially constructed three-electrode spectroelectrochemical cell (Fig. 2) containing an electrolyte composed of water solution of 10 mM sodium ascorbate (Asc), 200 µM dichlorophenoloindophenol (DCPIP) and 30 mM Bis–Tris pH 7.0 buffer. The electrodes were connected to the Autolab PGSTAT204 potentiostat. The counter electrode (CE) was a platinum wire, and the reference electrode (RE) was Ag/AgCl filled with 3 M KCl (+ 220 mV vs SHE). However, all the data presented in this paper were recalculated and are shown vs standard hydrogen electrode (SHE). To induce photocurrent in chronoamperometric measurements, the PSI-FTO working electrode (WE) was illuminated by the 685-nm LED characterized by the ~ 24-nm-wide spectral band (FWHM) and illumination density of 5.8 mW/cm^2^. Typically, the illumination time was 30 s and the dark adaptation time was 50 s, except for the potential dependence measurements for which the dark adaptation time was extended to 200 s after each change of the applied potential. For the latter measurements, the value of photocurrent was read out as a peak value of the photocurrent after baseline dark current subtraction (see supplementary information for the detailed description of the procedure).

When stated that data were obtained at open circuit potential (OCP), the OCP was measured by the potentiostat in the darkness and then applied to WE to keep zero dark current between WE and CE.

Measurements of the photocurrent action spectra were carried out with a photoelectric spectrometer (Instytut Fotonowy) and the same spectroelectrochemical cell as used for the chronoamperometric measurements. The illumination was via a monochromator from a xenon arc lamp, and the light intensity was constantly monitored for correction of the photon flux at the sample. The applied potential was + 100 mV (OCP in darkness) for the cathodic photocurrents and + 520 mV for anodic. At each wavelength, the light was turned on for 10 s and then turned off for 10 s. For analysis, the average photocurrent from the last 2 s of illumination was extracted after the dark photocurrent baseline correction (see the supplementary information).

In most of the experiments, the oxygen was present in the electrolyte and in the atmosphere inside the spectroelectrochemical cell. However, we have performed a control experiment with the electrolyte bubbled with argon for ~ 10 min and the spectroelectrochemical cell being continuously flushed with argon during the measurements. No significantly different photocurrent was observed under the deoxygenated conditions.

### Estimation of internal and external quantum efficiencies

Internal quantum efficiency (IQE) was estimated on the basis of the LED emission spectrum, the PSI absorption spectrum in solution scaled to fit the amplitude of *Q*_y_ absorption band of PSI deposited on FTO, and typical values of the photocurrents generated for OCP. The IQE was defined as the ratio of generated electron flux to the flux of the absorbed photons. External quantum efficiency (EQE) was defined as the ratio of generated electron flux to the flux of the incident photons. The LED emission spectrum was measured using an Avantes Hero fiber optics spectrometer calibrated for the CCD sensitivity. The PSI absorption spectrum was measured in buffered (20 mM Bis–Tris pH 7.0) solution with detergent (0.03% *β*-DM) using a Hitachi U-2800A spectrophotometer. See supplementary information for detailed description of the IQE and EQE estimation.

### Transient absorption measurements

Time-resolved absorption data were collected using 1 kHz pump-probe Helios system (Ultrafast Systems), described in detail previously (Szewczyk et al. 2018). Shortly, the excitation pulses (800 nm, ~ 200 fs wide, FWHM) were generated in the Ti:Sapphire oscillator (Mai-Tai, Spectra Physics), then amplified in the regenerative amplifier (Spitfire Ace, Spectra Physics), and tuned in the optical parametric amplifier to 650 nm (Topas Prime, Spectra Physics). The probe white-light continuum was generated in the sapphire crystal. The pump energy was approximately 50 nJ, and the spot size of the beams at the sample—about 200 µm.

The PSI-FTO electrode was placed in a home-build spectroelectrochemical cell dedicated to transient absorption measurements different from that used for the current measurements (see Fig. 7c and d. Next, the cell was filled with approximately 3 ml of 30 mM Bis–Tris pH 7.0 solution, and the PSI-FTO electrode, together with the RE and CE were connected to the Autolab PGSTAT204 potentiostat. The pump-probe experiments were performed consecutively at two biases applied to WE, − 180 and + 620 mV, each time after about 10 min of the system adaptation to a new potential. To ensure sample relaxation between consecutive excitation pulses, the cell was continuously moved during the experiment by a mechanical motion controller in two directions perpendicular to each other and to the excitation beam.

The time window of the experiments was 2.6 ns. For each potential, four 15-min scans of probe light intensity were collected alternately from the smallest to the largest and from the largest to the smallest delay times between the pump and probe pulses. Under the described conditions, typical absorbance changes at the maximum of the signal was above 1 mOD. Obtained data were corrected for the spectral chirp of white-light continuum and the spectral background in the SurfaceXplorer software (Ultrafast Systems). Global analysis of the results was performed using Glotaran software (Snellenburg et al. 2012).

## Results and discussion

### Cathodic photocurrent at open circuit potential

**Fig. 3:**
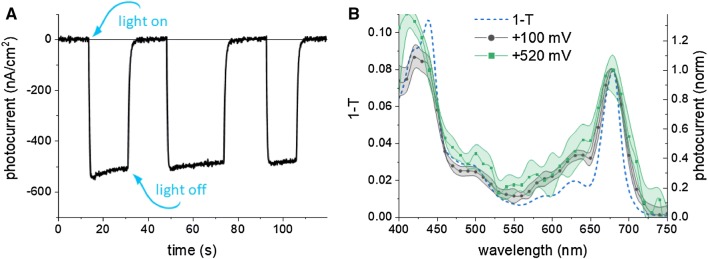
**a** Chronoamperometric data of PSI-FTO WE in three-electrode system recorded for OCP =  + 100 mV showing a net cathodic photocurrent. The electrolyte was water solution of 30 mM Bis–Tris buffer, pH 7.0 containing 200 μM DCPIP and 10 mM sodium ascorbate. 685-nm LED illuminated the WE with a power density of 5.8 mW/cm^2^ at the electrode surface. **b** Comparison of 1-T spectrum (*T* = 10^−A^, T—transmission, A—absorbance) of PSI in solution, photocathodic action spectrum of PSI-FTO WE recorded for OCP =  + 100 mV, and photoanodic action spectrum of PSI-FTO WE recorded at + 520 mV. The standard errors of the photocurrent values in the action spectra is presented

Figure 3a shows typical chronoamperometric data recorded for the PSI-FTO WE in the spectroelectrochemical cell shown in Fig. 2 yielding the photocurrent of ~ 500 nA/cm^2^. These data were recorded after standard single electrodeposition step in which ~ 5 monolayers of PSI were formed. In a control experiment (not shown), we checked that the photocurrent increases proportionally to the number of the electrodeposition steps. The data were recorded for OCP =  + 100 mV, which was the potential applied to WE blocking any dark current but not photocurrent. The value of OCP is the redox potential of the mixture of electrolyte components used and thus is dictated by the concentration, initial redox states and midpoint potentials (*E*^0^) of DCPIP (added to the solution in the oxidized form) and ascorbate (added in the reduced form). As expected, after mixing of the reduced ascorbate (10 mM) and oxidized DCPIP (200 µM), the ascorbate fully reduces DCPIP and OCP of + 100 mV is established, which is a value intermediate between *E*^0^ of ascorbate (+ 60 mV) and DCPIP (+ 217 mV) (Fig. S5). Full reduction of DCPIP by excess of ascorbate is evidenced by complete disappearance of blue color of the solution of oxidized DCPIP after addition of ascorbate

**Fig. 4:**
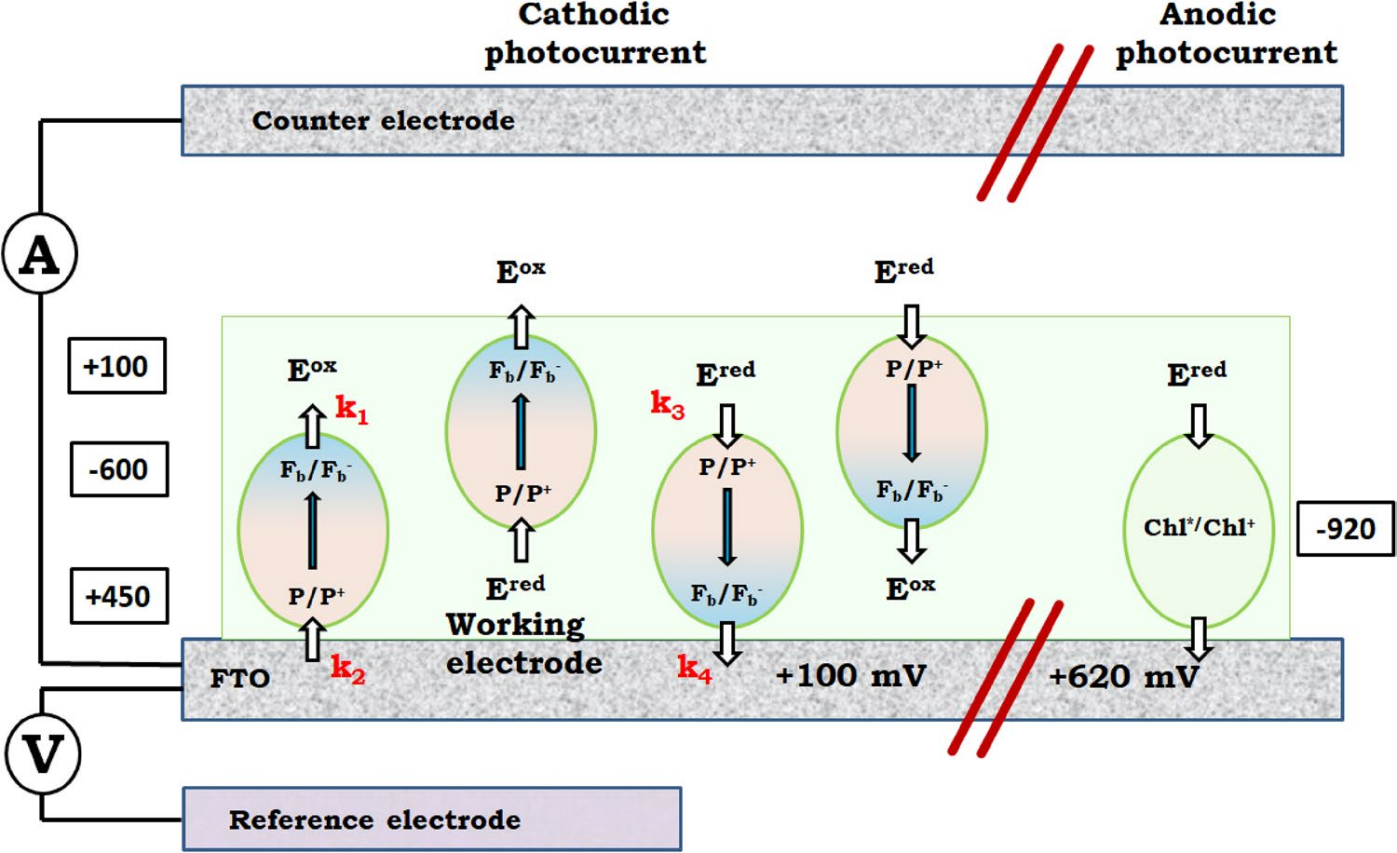
Interactions of PSI complexes with FTO conducting glass and electrolyte. Left side, potential of + 100 mV applied to WE, net cathodic photocurrent: two specific orientations of PSI particles are shown with either donor or acceptor PSI side facing FTO; PSI complexes interacting both directly and via electrolyte with the FTO substrate are shown. Right side, potential of + 620 mV applied to WE, net anodic photocurrent: due to electrochemical oxidation of P700, orientation of PSI complex does not play a role. Redox potential of electrolyte (+ 100 mV) as well as redox midpoint potentials of P700/P700^+^ (+ 450 mV), F_b_/F_b_^−^ (− 600 mV), and Chl*/Chl^+^ (− 920 mV) are shown in the rectangles next to respective redox pairs. *E*^ox^ and *E*^red^ denote oxidized and reduced mediators, respectively

The photocurrent shown in Fig. 3 is negative or cathodic, meaning that the electrons are transferred from the FTO substrate to PSI, then to electrolyte and to CE (Fig. 4). Such photocurrent direction is expected since the electrodeposition privileges orientation of PSI with its donor side facing the FTO surface (Fig. 1). With such orientation, the P700 dimers from the first PSI monolayer interacting directly with the FTO are close enough to the substrate to promote direct electron transfer from FTO to P700^+^ which is possible due to the suitable potentials of FTO and P700/P700^+^ (Figs. S5 and 4). The PSI complexes forming layers more distant from the FTO may accept electrons from the FTO via the electrolyte mediators and/or from the PSI complexes forming layers closer to the FTO surface (see below).

Analysis of the redox potentials of the electrolyte, P700/P700^+^, and F_b_/F_b_^−^, as well as the OCP potential of the WE leads to the conclusion that the opposite direction of the photocurrent, from the PSI to FTO is also possible, but apparently less efficient (Figs. S5 and 4). Such anodic photocurrent most likely coexists with the cathodic one. Its lower efficiency may come from (1) the preferential donor-side orientation of the majority of the PSI complexes and/or (2) lower values of the molecular rate constants *k*_3_ and *k*_4_ than *k*_1_ and *k*_2_, respectively (Fig. 4). Indeed, it is known that the acceptor side of PSI is most easily accessible to exogenous electron acceptors (Izawa 1980; Böhme et al. 1971; Izawa et al. 1973; Trubitsin et al. 2014) which implicates that *k*_1_ > *k*_3_.

To prove an engagement of the PSI in generation of the photocurrent in the PSI-based electrodes, the action spectrum or photocurrent vs. illumination wavelength can be presented (Yu et al. 2015; Stieger et al. 2016b). In Fig. 3b, a cathodic current action spectrum for the PSI-FTO WE is shown and compared with the 1-T spectrum of the PSI-buffered solution. One can see that the two spectra are very similar to each other unlike the respective spectra published before (Yu et al. 2015; Stieger et al. 2016b). It is worth to note at this point that such a similarity indicates only that the antenna PSI pigments are responsible for the light absorption leading to the generation of photocurrent but says nothing on the intactness of electron transfer in the PSI reaction center. In other words, it does not prove that the electrons are accepted from the FTO substrate by P700^+^ and transferred to the CE via F_b_^−^. This is because the absorption spectrum of PSI is dominated by the antenna Chls. On the other hand, a deviation of the action spectrum from the 1-T (or absorbance) spectrum is indicative of specific modifications in the functioning of the PSI on the level of antenna. For example, in Yu et al. (2015) a large blue-shift of both *Q*_y_ and especially Soret band of Chls has been observed, most likely reflecting extraction of Chls from the PSI deposited on a substrate and exposed to the acetonitrile solvent. From our measurements for OCP, we conclude only that all the photocurrent originates from the PSI. Below we present arguments that the photocurrent generation is related to the light-induced formation of the state P700^+^F_b_^−^.

### Cathodic and anodic photocurrent at different potentials

**Fig. 5:**
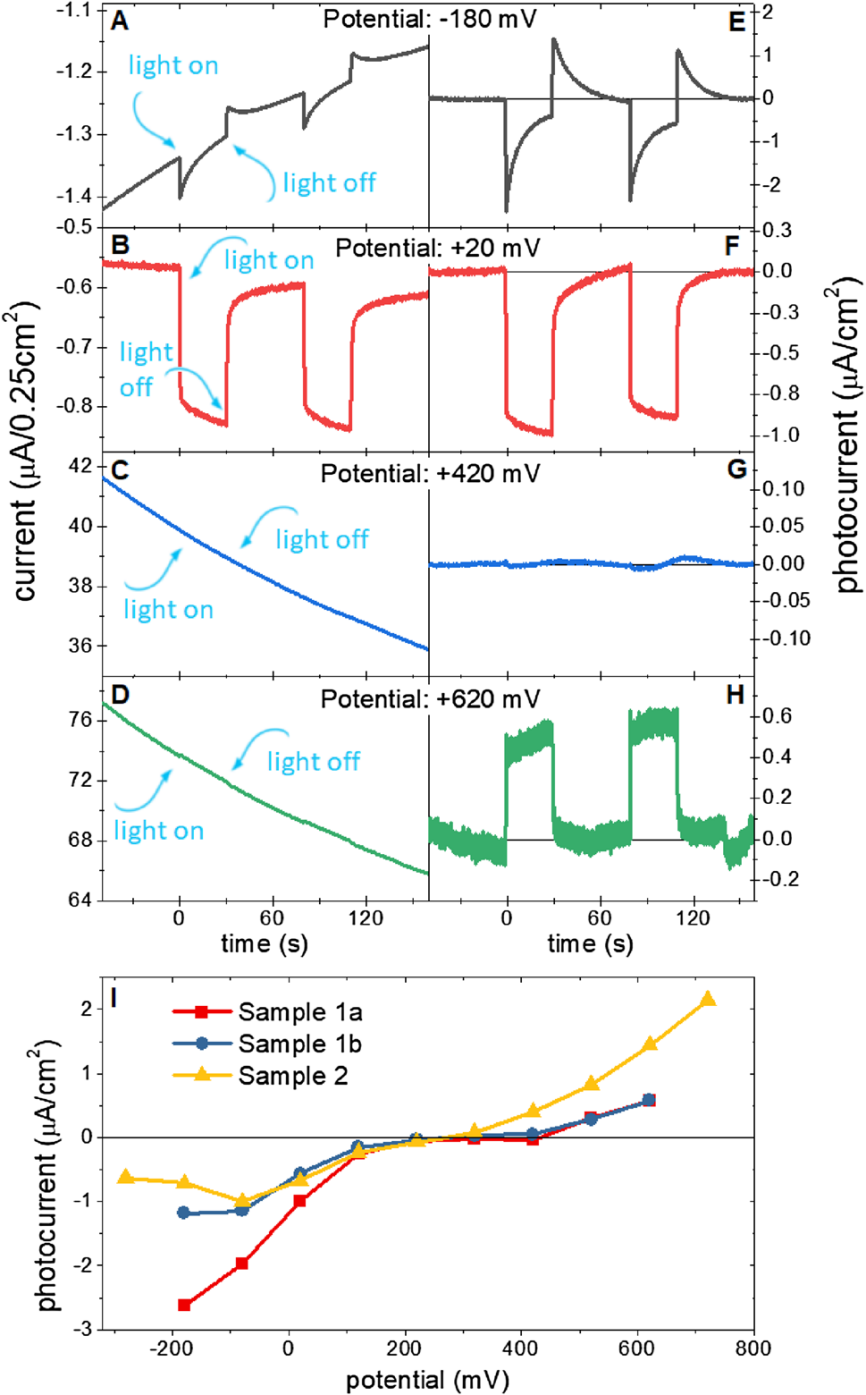
Results of current measurements at a range of potentials applied to PSI-FTO WE. **a**–**d** raw chronoamperometric data recorded for PSI-FTO WE at four selected potentials; **e**–**h** data from panels **a**–**d** after subtraction of baselines caused by dark current; (**i**) three sets of photocurrent vs potential measurements; sequences of applied potentials were the following: Sample 1a—+ 120, − 180, …, + 620 mV, Sample 1b—+ 620, …, − 180 mV; Sample 2—each point is an average of points from three curves collected consecutively with the following sequences of applied potentials: 1. Curve—+ 120, …, + 720 mV, 2. Curve—+ 720 mV, …, − 280 mV, 3. Curve—− 280, …, + 120 mV

Application of potentials different from the OCP induced large dark currents (Fig. 5a–d; smaller negative ones at low potentials due to the reduction of oxidized ascorbate and much more pronounced positive ones at high potentials due to the oxidation of reduced DCPIP and/or ascorbate at the electrode surface) which were treated as baselines and subtracted from the measured traces to extract pure photocurrents (see supplementary information). The result of such subtraction is shown in Fig. 5e–h. Figure 5i shows dependence of extracted photocurrents (measured soon after switching on the light) on the potential applied to WE. As expected, application of the potentials lower than OCP, down to ~ − 180 mV resulted in increase of the cathodic photocurrent to 1–2.5 µA/cm^2^. The photocurrent amplitude at low potentials depends on the sequence of applied bias (see caption to Fig. 5i). Particularly large photocurrent was measured when just after the measurement for OCP, the photocurrent was measured at − 180 mV (Sample 1a). When the same sample was adapted to low potentials for longer times, the cathodic photocurrent at negative potentials was about twice smaller (Sample 1b). Moreover, for samples slowly adapted to low potentials, the photocurrent measured at − 180 and − 280 mV gets smaller with decreasing potentials (Sample 2). These two features may be explained by a slow reduction of soluble mediator in the electrolyte in the proximity of the WE and thus its inability to accept electrons from PSI.

At potentials ranging from ~  + 200 to ~  + 400 mV the photocurrent was close to zero and at higher potentials, a positive or anodic photocurrent was measured (Fig. 5i). As one can see, the anodic photocurrent increases systematically with potential of up to + 720 mV. To reveal the origin of the anodic photocurrent, an action spectrum at + 520 mV was measured (Fig. 3b). As one can see, its shape is very similar to that one measured for OCP =  + 100 mV and to the 1-T spectrum of the PSI measured in solution. This proves that also anodic photocurrent originates from PSI.

The anodic photocurrent means that an electron is injected from PSI to FTO. One possible candidate to inject electrons to the FTO is F_b_^−^ (Fig. 4). However, as shown below, at potentials much more oxidizing than + 450 mV, which is the redox midpoint potential of the P700/P700^+^ pair, the PSI primary donors becomes permanently oxidized and the observed photocurrent cannot result from F_b_^−^ to FTO electron transfer since the natural P700-F_b_ → P700^+^-F_b_^−^ charge separation is inhibited. Therefore, we propose that the anodic photocurrent, at least at the most oxidizing potentials originates from excited antenna Chls (Fig. 4). It was demonstrated that at high potentials (+ 650 nm) even the PSI antenna Chls being in the ground state may undergo electrochemical oxidation (Nakamura et al. 2004). Therefore, it is justified to expect that the excited Chls (Chls*) which have got very negative midpoint redox potential (− 920 mV) (Zhang et al. 2016) will be oxidized by a high-positive potential applied to the FTO despite short excited state lifetime of antenna Chls (of the order of 30 ps; (Gobets et al. 2001)), orders of magnitude shorter than the lifetime of F_b_^−^ (millisecond range; Vassiliev et al. 1997; Kurashov et al. 2018). This hypothesis is supported by recent observation that excited Chls in the PSII antenna may efficiently donate their electrons to the highly oxidizing acceptor rather than transfer their energy towards the reaction center (Zhang et al. 2016).

Even more likely is that the electrons are injected to the FTO substrate from a fraction of Chls which are energetically uncoupled from the PSI reaction center. Such Chls are commonly observed in virtually all the PSI preparations (usually a few percent of all PSI Chls are uncoupled Chls; (Szewczyk et al. 2020; Gobets et al. 2001; Savikhin et al. 1999; Holzwarth et al. 2005; Quiniou et al. 2015) and were also detected in the PSI-FTO electrodes (Szewczyk et al. 2017, 2018)). The excited state lifetime of the uncoupled Chls is extended to ~ 0.5–5 ns—one to two orders of magnitude longer than the excited state lifetime of Chls in the fully intact PSI. Moreover, the uncoupled Chls are preferentially located at the periphery of PSI complex (Szewczyk et al. 2020). These circumstances make the uncoupled Chls more likely candidates for electron injection to the FTO than the majority of Chls well coupled to the reaction center. Different amplitudes of the anodic photocurrents for Sample 1 and Sample 2 in Fig. 5i could be due to different amount of the uncoupled Chls in these two electrodes.

### Exogenous electron donors and acceptors for PSI

As shown in Fig. 4, the redox potential of the buffered electrolyte composed of 200 µM DCPIP and 10 mM ascorbate is + 100 mV. However, the symbols of oxidized and reduced electrolyte, *E*^ox^ and *E*^red^, do not specify the chemical nature of electron acceptors and donors involved in electron exchange between the electrolyte and PSI. The mixture of DCPIP and ascorbate is a complex electrolyte because (1) both DCPIP and ascorbate undergo two-electron redox reactions accompanied by proton release or uptake (Iyanagi et al. 1985), (2) DCPIP is fully reversible redox compound (Izawa 1980), whereas ascorbate is regarded as a sacrificial electron donor (Ciesielski et al. 2008, 2010a) although, in principle, oxidation of ascorbate is also reversible (Sapper et al. 1982; Iyanagi et al. 1985).

**Fig. 6:**
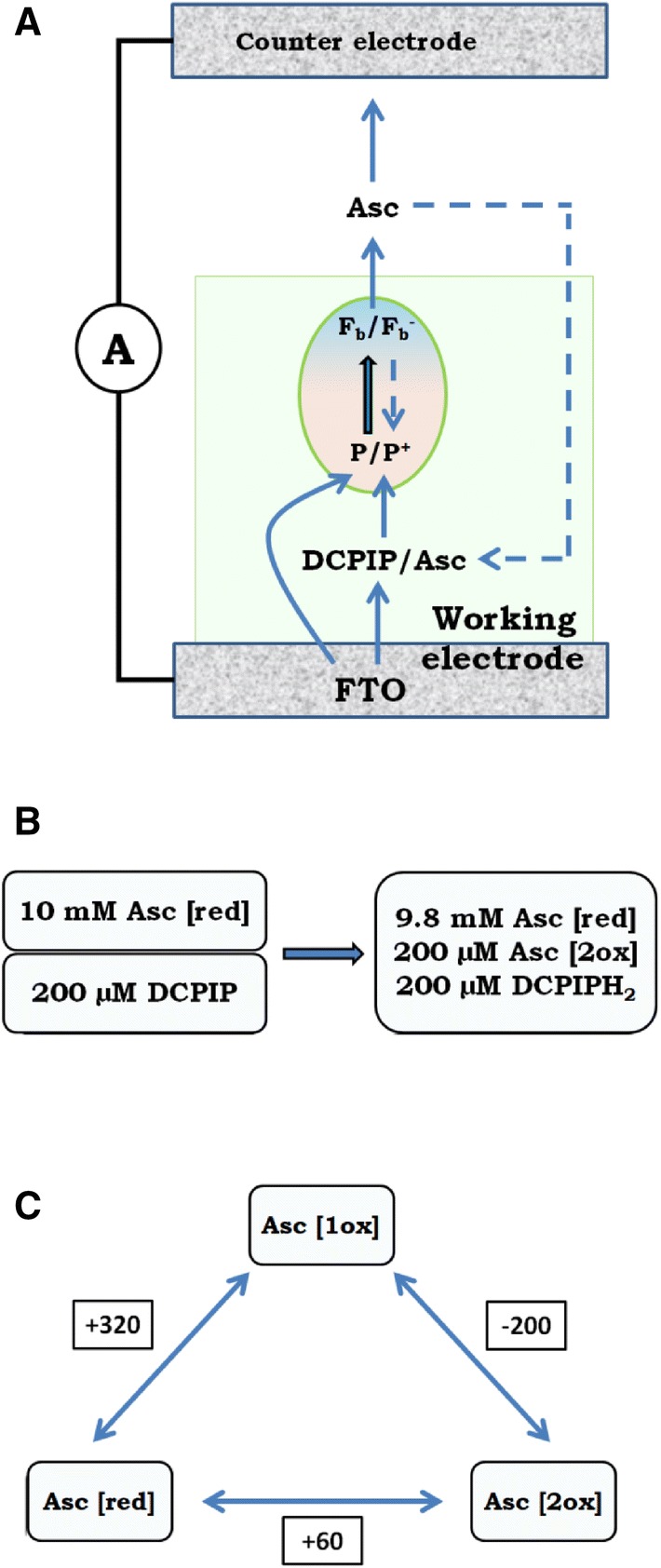
**a** Proposed engagement of ascorbate and DCPIP in electron transfer inside the PSI-FTO-based photoelectrochemical cell. Continuous lines represent linear electron transfer from WE to CE at WE potentials equal OCP or close to OCP. The dashed lines represent dissipative PSI internal and external back electron transfers. **b** Expected effect of mixing of 10 mM ascorbate and 200 µM DCPIP. **c** Three reversible redox forms of ascorbate and midpoint redox potentials of the three redox pairs (Adapted from Sapper et al. 1982; Asc[red]—ascorbate, Asc [1ox]—semihydroxyascorbate radical, Asc[2ox]—dehydroxyascorbate)

Figure 6a shows a simplified scheme of proposed dominating pathways of the electron transfer inside the PSI-FTO-based photoelectrochemical cell. The major point is that ascorbate is an electron acceptor from F_b_^−^. In principle, both DCPIP (Izawa 1980) and ascorbate (Trubitsin et al. 2014; Miyake and Asada 1992; Mano et al. 2004; Ivanov et al. 2005; Hiyama and Ke 1971) may be both electron donors and acceptors for PSI (being in the state P700^+^F_b_^−^) depending on their redox states (compare midpoint potentials of DCPIP/DCPIPH_2_, Asc, P700/P700^+^, and F_b_/F_b_^−^ in Fig. S5). However, the 50-fold excess of reduced form of ascorbate in the solution leads to complete reduction of DCPIP (see above) and the reduced DCPIP (DCPIPH_2_) may only be an electron donor to P700^+^. Expected concentrations of oxidized and reduced forms of ascorbate and DCPIP after mixing them in electrolyte solution is shown in Fig. 6b. We conclude that the only species able to accept electrons from F_b_^−^ are oxidized forms of ascorbate: Asc[1ox] (semidehydroascorbate) and/or Asc[2ox] (dehydroascorbate; Fig. 6c). It is in contrasts to what was proposed before for a similar system containing spinach PSI multilayer on a gold substrate (Ciesielski et al. 2010a) (see Table 1), where DCPIP was proposed to be both electron donor and acceptor for PSI despite of similarly large as in the present study, 20-fold excess of reduced ascorbate. In Ciesielski et al. (2010a), none function was attributed to ascorbate, except for the initial reduction of DCPIP. The full reduction of DCPIP together with the high concentration of the reduced ascorbate (Fig. 6b) do not allow conclusion on which of these two mediators is dominating electron donor to P700^+^. Actually, it is even possible that the dominating electron transfer to P700^+^ occurs directly from FTO or from F_b_^−^ of adjacent PSI complex. The argument for that comes from the transient absorption studies presented below

**Table 1:** Comparison of results obtained for two PSI-based spectroelectrochemical cells with electrolyte composed of DCPIP and ascorbate

	Ciesielski et al. (2010a)	This work
Protein	PSI from spinach	PSI from cyanobacteria *Synechocystis* sp. PCC 6803
Substrate	Gold	FTO conducting glass
Deposition method	Drop-casting + vacuum drying	Drop-casting + electrodeposition + overnight drying at 4 °C
Number of PSI monolayers	80–160^a^	~ 5
Thickness of PSI layer	1–2 µm^b^	~ 60 nm^a^
Absorbance of PSI layer on the substrata at maximum of *Q*_y_ band (~ 679 nm)	–	0.030 ± 0.005
Electrolyte	5 mM DCPIP, 100 mM Na-ascorbate	200 µM DCPIP, 10 mM Na-ascorbate
Number of electrodes	2 (WE + CE/RE)	3 (WE + CE + RE)
Illumination	White light, 95 mW/cm^2^	Red light (685 ± 10 nm), 5.8 ± 0.4 mW/cm^2^
Maximal cathodic photocurrent	− 7 µA/cm^2^ @ 0 V (OCP = + 70 mV)	0.5 µA/cm^2^ @ OCP = + 100 mV − 2.5 µA/cm^2^ @ − 180 mV
IQE	–	0.37 ± 0.11%
EQE	0.001%	0.016%
Action spectrum	No	Yes (Fig. 3)

^a^Value obtained assuming the diameter of PSI and the thickness of PSI monolayer of 12 nm

^b^Measured by profilometry

Figure 5i indicates that at the WE potential of ~ − 200 mV, the photocurrent starts to decrease with decreasing potentials. On the other hand, − 200 mV is the midpoint potential of Asc[1ox]/Asc[2ox] (Fig. 6c; Sapper et al. 1982; Iyanagi et al. 1985). This coincidence suggests that Asc[2ox] is the main electron acceptor from F_b_^−^, and that Asc[2ox] present in the electrolyte layer adjacent to WE may be efficiently electrochemically reduced to Asc[red] (via the state Asc[1ox], which is much easier to reduce (E^0^ =  + 320 mV) than the state Asc[2ox]) by the negative potential applied to WE

### Internal and external quantum efficiencies of the PSI-FTO electrode

Figure 6a indicates that for the OCP, in addition to a linear electron transport from the WE to the bulk generating the cathodic photocurrent, the exogenous redox molecules may take part in the external back electron transfer from F_b_^−^ to P700^+^ reducing the photocurrent (long-dashed arrow). Furthermore, internal back electron transfer competing with electron delivery to P700^+^ and uptake from F_b_^−^ may lead to further photocurrent limitation (short dashed arrow), similarly as photoanodic current caused by possible opposite orientation of minority of the PSI complexes on FTO (Fig. 4). To estimate an effect of these counterproductive electron transfer pathways, the internal quantum efficiency (IQE) of our system has been estimated in a way described in “Materials and methods” and in the supplementary information (Eq. S4). The obtained value of 0.37 ± 0.11% (for OCP) is ~ 20 times higher than that published for a similar system based on the electrodes composed of truncated spinach PSI deposited on a functionalized gold (Ciesielski et al. 2010b). At more negative potentials (~ -180 mV) photocurrent may increase even fivefold (Fig. 5i) which translates linearly into the IQE. The external quantum efficiency (EQE) (Eq. S5) corresponding to IQE = 0.37 ± 0.11% equals 0.016%. This value is low due to the low absorbance of the thin PSI layer on FTO. It should not be compared directly with EQE from the literature (for example in Ciesielski et al. 2010a, Table 1) since specific red light was used as a source of illumination (Fig. S2), largely overlapping with the absorption spectrum of PSI (Figs. S1 and S2), and not the standard white light

### Pontentiostatic control of the redox state of P700

**Fig. 7:**
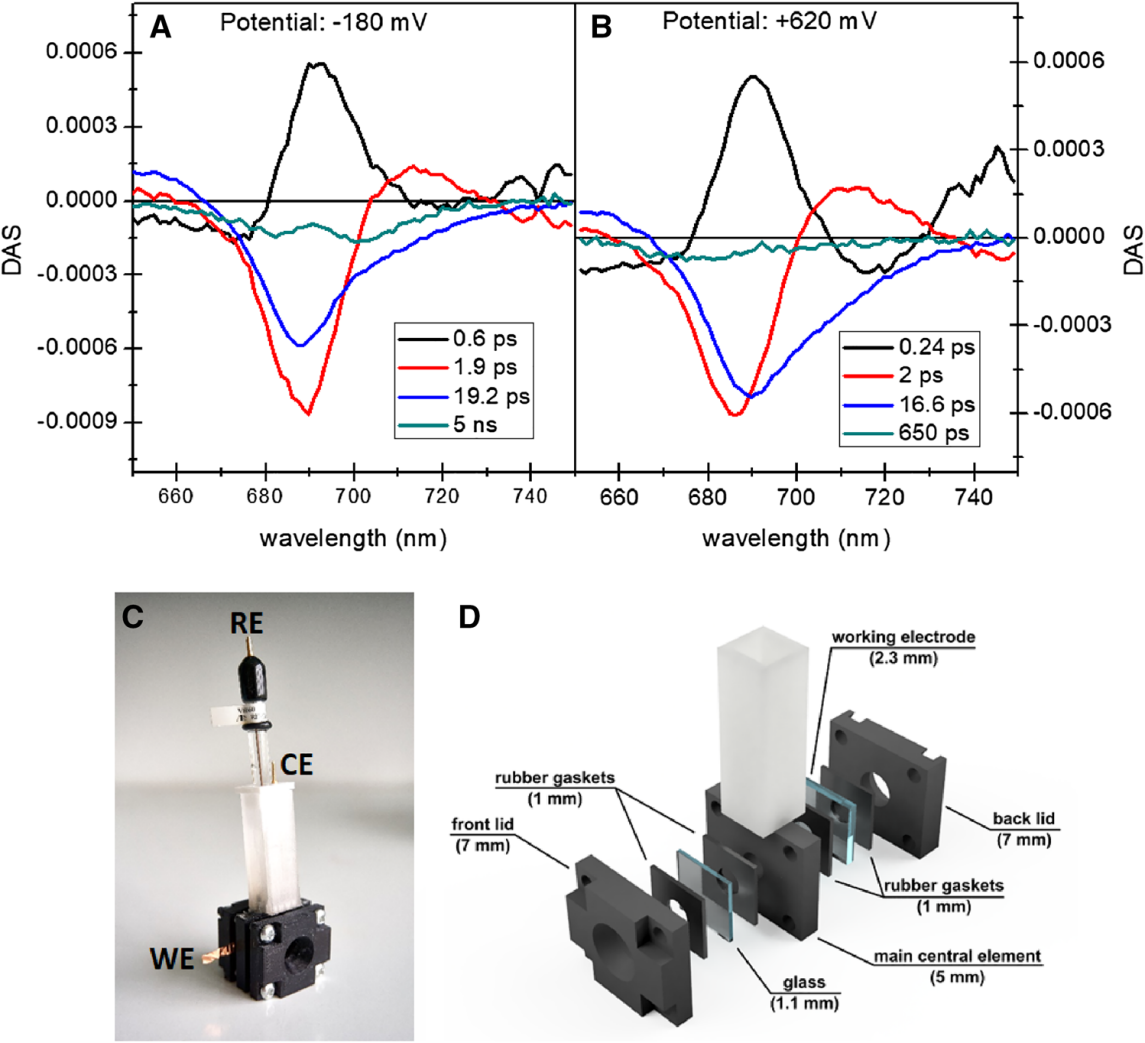
Results of time-resolved absorbance measurements of PSI-FTO WE (**a**—at − 180 mV bias, **b**—at + 620 mV bias) and three-electrode spectroelectrochemical cell dedicated to these measurements (**c**—assembled, **d**—exploded cell). Excitation wavelength was 650 nm

To confirm that the very high WE potential inactivates natural electron transport inside PSI (by oxidation of P700) and thus to reinforce the hypothesis that the anodic photocurrent shown in Fig. 5 originates from the photooxidation of the antenna Chls, ultrafast time-resolved absorption measurements were performed on the PSI complexes deposited on the FTO. Figure 7a and b compares results of global analysis (Decay Associated Spectra, DAS) of the data collected at two extreme potentials applied to the PSI-FTO WE: − 180 mV at which all P700 dimers are expected to be in their neutral state in darkness (open PSI), and + 620 mV at which most of the P700 dimers are expected to be in their oxidized state, P700^+^, in darkness (closed PSI). The DAS spectra in these two figures are very similar to those obtained for, respectively, chemically open and closed PSI (from the same cyanobacteria) in solution (Savikhin et al. 2000). Light pulses centered at 650 nm are expected to excite vibronic states coupled with *Q*_y_ transition of Chls *a* as well as pure *Q*_y_ electronic states of Chls *a* of particularly low-site energies. Therefore, the subpicosecond component in Fig. 7a and b, is assigned to a mixture of internal relaxation in Chls (appearance of the stimulated emission at ~ 690 nm) and excitation energy transfer to the lower energy Chls. The ~ 2 ps component is due to both fast excitation decay and excitation energy transfer from bulk (685–690 nm) to red Chls (~ 710 nm), and the ~ 18-ps component is due to a decay of the excited state equilibrated over the bulk and red Chls. All these components are similar at both potentials. The essential difference is observed for the slowest components. For the open PSI, the shape of this spectrum is typical for the P700^+^–P700 ΔA spectrum (Savikhin et al. 2000, 2001) with characteristic minima at ~ 680 and ~ 700 nm. The 5-ns lifetime of this DAS is significantly longer than the 2.6-ns time window of the experiment and, therefore, may be treated as a non-decaying component. This means, that under the reducing conditions, the PSI remains in the open state and each excitation generates a state P700^+^. Under the oxidizing potential, almost no signal at 700 nm remains long after excitation. Instead, the lifetime (650 ps) and blue-shifted maximum (to ~ 680 nm) of the slowest DAS is characteristic for the uncoupled Chls in the PSI deposited on FTO (Szewczyk et al. 2017, 2018). One may conclude that under the oxidizing conditions practically all the PSI complexes remain in the closed state. This observation bears important consequences. First, it demonstrates that the high-positive potential applied to the WE may efficiently oxidize not only the first PSI monolayer interacting directly with FTO but also PSI complexes more distant from the substrate. It is worth to note that the buffer solution filling the spectroelectrochemical cell for the transient absorption measurement did not contain any mediators. Thus, electron transfer from P700 of the distant PSI to FTO, induced by high the FTO potential, has to occur by electron hopping between PSI complexes. Such a possibility indicates that the photocurrent may also flow by hopping between the PSI complexes with no involvement of the mediators. Finally, the full oxidation of P700 in all PSI deposited on the FTO excludes a possibility that the anodic photocurrent observed at the high-positive potentials (Fig. 5) is generated as a result of the formation of the state P700^+^F_b_^−^ and injection of an electron from F_b_^−^ to FTO in a minor fraction of PSI oriented with the acceptor side facing FTO (Fig. 4). This reasoning supports the hypothesis that the anodic photocurrent at the very positive potentials originates from the photooxidation of the excited Chls in the antennae.

## Conclusion

In this paper, we demonstrate a significant photovoltaic activity of the biohybrid electrodes containing PSI particles which have been proven to be functionally highly intact at the level of ultrafast processes: excitation energy transfer and primary charge separation (Szewczyk et al. 2017, 2018). Apparently, the IQE of 0.37% for the OCP is not limited by inefficient charge separation but it may be caused by the internal back electron transfer inside the PSI (both in the fully functional complexes allowing formation of the state P700^+^F_b_^−^, and in functionally impaired complexes showing charge recombination between P700^+^ and intermediate PSI electron acceptors (Kurashov et al. 2018) or by the external back electron transfer promoted by exogenous redox molecules. Two opposite contributions of the photocurrent were identified: the cathodic one—seen at the reducing conditions and originating from the P700^+^F_b_^−^ charge-separated state, and the anodic one observed at the oxidizing conditions—originating most likely from the photooxidized antenna Chls. Our finding that the electrical bias applied to the PSI-FTO electrode fully controls the redox state of P700 may be used in the future to characterize the energy and electron transfer reactions in the immobilized PSI proteins and the effect of the P700 redox state on these reactions. Such studies on different PSI biohybrid electrodes are necessary to prove the usefulness of the PSI application in such systems and to optimize their functionality.

## Electronic supplementary material

Below is the link to the electronic supplementary material.

Supplementary file 1 (DOCX 367 kb)
