# Supplementary material for: Photovoltaic activity of electrodes based on intact photosystem I electrodeposited on bare conducting glass
Source: Photosynth Res. 2020 Feb 20;144(1):1–12. doi: 10.1007/s11120-020-00722-1 (PMC7113217; doi:10.1007/s11120-020-00722-1)
Supplement: Supplementary file 1 — (DOCX 367 kb) [file 11120_2020_722_MOESM1_ESM.docx]

**Supplementary Information**

Photovoltaic activity of electrodes based on intact photosystem I electrodeposited on bare conducting glass

Sebastian Szewczyk, Rafał Białek, Gotard Burdziński, Krzysztof Gibasiewicz*

Faculty of Physics, Adam Mickiewicz University in Poznań, ul. Uniwersytetu Poznańskiego 2, 61-614 Poznań, Poland

*corresponding author

# IQE and EQE estimation

For estimation of IQE and EQE values following information is necessary:

- incident photon flux distribution (IPFD; spectrum of incident light),
- photocurrent generated from the unit area of photoelectrode.

Moreover, for IQE estimation, the absorption spectrum of the active element of the working electrode is necessary to calculate the absorbed photon flux distribution (APFD). For this, the absorption spectrum of PSI in buffered solution was used and properly scaled to meet the absorbance of PSI on the FTO surface (Figure S1). It was so, as the absorption spectrum in solution was measured with much higher signal-to-noise ratio than that on FTO.

Figure S1 Absorption spectrum of PSI in buffered aqueous solution scaled to match an average absorbance of PSI on FTO at the maximum around 680 nm. The absorbance at maximum around 680 nm used for further calculations was 0.030±0.005.

To obtain the IPFD, the spectrum of the LED was measured as a number of counts at the detector as a function of wavelength. Subsequently, the spectrum was scaled using known 5.8±0.4 mW/cm^2^ total irradiation and energy of photon of certain wavelength:

|  | $E=h\frac{c}{\lambda}$ |  |
| --- | --- | --- |

where: $E$ – energy of photon, $h$ – Planck constant, $c$ – speed of light, $\lambda$ – wavelength.
This procedure led to the IPFD spectrum as in Figure S2.

To obtain the APFD, equation S2 was used:

|  | $APFD\left( \lambda\right)=IPFD\left( \lambda\right)\left( 1-{10}^{-A(\lambda)} \right)$ |  |
| --- | --- | --- |

where: $\lambda$ – wavelength, $A(\lambda)$ – absorbance.
This led to the APFD curve in Figure S2.

Figure S2 Spectra of incident and absorbed photon fluxes.

To obtain the electron flux, $n_{electr}$, equation S3 was used. The value of the photocurrent density used in calculations was 500±100 nA/cm^2^.

|  | $n_{electr}=\frac{J}{e}$ |  |
| --- | --- | --- |

where: $J$ – photocurrent density, $e$ – elementary charge.

And finally for IQE and EQE calculations, equations S4 and S5 where used.

|  | $IQE=\frac{n_{electr}}{\int APFD\left( \lambda\right)d\lambda}100\%$ |  |
| --- | --- | --- |
|  | $EQE=\frac{n_{electr}}{\int IPFD\left( \lambda\right)d\lambda}100\%$ |  |

# Baseline correction for action spectra

Data obtained directly from action spectrum measurements contained baseline dark current and photocurrent signal from light on-light off cycles for different wavelengths (Figure S3, blue circles). To get only the information about the photocurrent the baseline correction procedure was applied. First, data was smoothed using Savitzky-Golay filter (Figure S3, orange line). Next, the current from the last 2 seconds of the light off period was chosen (Figure S3, red circles) and used for interpolation for all other times giving the baseline (Figure S3, red line). Subsequently, baseline was subtracted from the original data for photocurrent extraction and action spectrum construction.


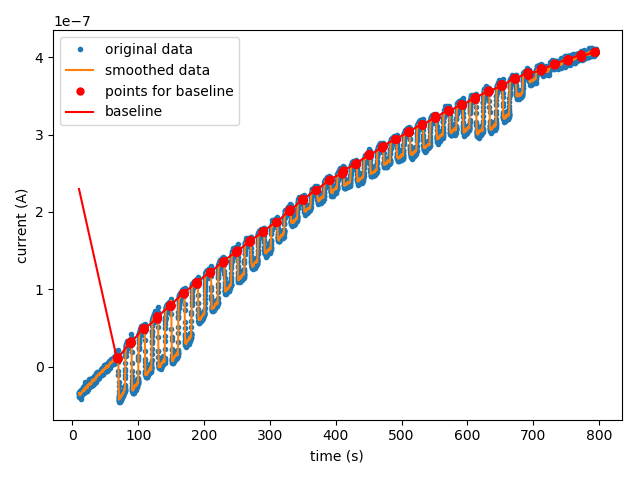


Figure S3 Baseline correction of data for action spectrum for sample cathodic action spectrum.

# Baseline correction for photocurrent vs potential measurements

Data obtained directly from photocurrent vs potential measurements contained baseline dark current and photocurrent signal from light on-light off cycles (Figure S4, black line). To get only the information about the photocurrent, the baseline correction procedure was applied. First, certain data points from the time, where the light was off were chosen (Figure S4, red circles). Next, polynomial curve was fitted to those points giving the baseline (Figure S3, red line). Subsequently, baseline was subtracted from the original data for photocurrent extraction.

Figure S4 Baseline correction for sample potential from photocurrent vs potential measurements.

# Redox midpoint potentials


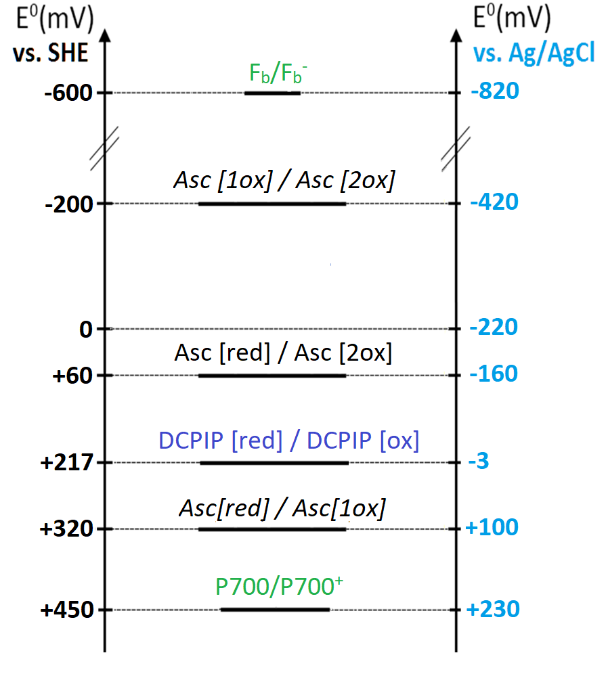


Figure S5 Comparison of the standard midpoint potentials of the electrolyte components, DCPIP (Kennet EC, Kuchel 2003; Efimov et al. 2014) and ascorbate (Sapper et al. 1982) as well as the primary and final electron transfer cofactors in PSI, P700 and F_b_ (Brettel and Leibl 2001; Nakamura et al. 2004).

References:

Brettel K (1997) Electron transfer and arrangement of the redox cofactors in photosystem I. Biochim. Biophys. Acta 1318:322–373

Efimov I, Parkin G, Millett ES, Glenday J, Chan CK, Weedon H, Randhawa H, Basran J, Raven EL (2014) A simple method for the determination of reduction potentials in heme proteins. FEBS Lett. 588(5):701-704

Kennet EC, Kuchel PW (2003) Redox reactions and electron transfer across the red cell membrane. IUBMB Life 55(7) 375-385

Nakamura A, Suzawa T, Watanabe T (2004) Spectroelectrochemical determination of the redox potential of P700 in spinach with an optically transparent thin-layer electrode. Chem. Lett. 33(6) 688-689

Sapper H, Kang SO, Paul HH, Lohmann W (1982) The reversibility of the vitamin C redox system: electrochemical reasons and biological aspects. Z. Naturforsch. 37c 942-946.
